# Better survival after breast-conserving therapy compared to mastectomy when axillary node status is positive in early-stage breast cancer: a registry-based follow-up study of 6387 Norwegian women participating in screening, primarily operated between 1998 and 2009

**DOI:** 10.1186/s12957-017-1184-6

**Published:** 2017-07-03

**Authors:** Olaf Johan Hartmann-Johnsen, Rolf Kåresen, Ellen Schlichting, Jan F. Nygård

**Affiliations:** 1 Cancer Registry of Norway, P.O. Box 5313, Majorstuen, 0304 Oslo Norway; 2 Department of Breast and Endocrine Surgery, Kalnes Hospital, Kalnes, Norway; 3 University of Oslo, Oslo, Norway; 4 Department of Breast and Endocrine Surgery, Oslo University Hospital, Oslo, Norway

**Keywords:** Breast conserving therapy, Mastectomy, Survival

## Abstract

**Background:**

Recent registry studies on early-stage breast cancer have shown better survival rates when women underwent breast-conserving therapy (BCT) compared with mastectomy (MTX). The aim of this study is to investigate women participating in screening, in all four stages of early breast cancer (T1N0M0, T2N0M0, T1N1M0, and T2N1M0), as to whether there is a survival benefit when women undergo BCT compared to MTX.

**Method:**

A cohort of 6387 women aged 50–69, with primary-operated breast cancer from January 1998 to December 2009, participating in screening and followed-up until the end of 2010. Life tables were calculated by stages (pT1N0M0, pT2N0M0, pT1N1M0, and pT2N1M0), surgery groups (BCT and MTX), and screening detection (first screening, later screening, or interval cancer). Cox regression was used to calculate hazard ratios (HR) between BCT and MTX in crude and adjusted analyses.

**Results:**

In stage T1N1M0, women who underwent MTX had an HR of 2.91 (95% CI 1.30–6.48) for breast cancer death compared to women who underwent BCT, after adjusting for screening detection, years of diagnosis, age at diagnosis, histology, grade, and hormone receptor status. For all other TNM categories of early breast cancer, there was no difference in survival.

10-year breast cancer-specific survival (BCSS) in T1N0M0 was 98% for women undergoing BCT and 96% for women undergoing MTX. 10-year BCSS in T1N1M0 was 97% for women undergoing BCT and 89% for women undergoing MTX.

**Conclusions:**

For women participating in screening, there is a benefit of BCT over MTX in stage T1N1M0. No such effects were observed in the other early stages of breast cancer.

## Background

Recent registry studies show better survival when women undergo breast-conserving therapy (BCT) compared with mastectomy (MTX) in early-stage breast cancer (T1-2N0-1M0). In 2013, Hwang et al. found better survival among patients undergoing BCT compared with MTX [1]. They suggested that differences in tumor biology (e.g., lymphovascular invasion or extranodal invasion) might contribute to survival differences between BCT and MTX.

In January 2014, Agarwal et al. published a paper corroborating the results of Hwang et al. [2]. They assumed that the difference in breast cancer-specific survival (BCSS) between BCT and MTX might be due to differences in adjuvant therapy regimes or tumor biology. In 2015, a Norwegian study corroborated the findings of the US studies [3].

One study has shown the benefit of BCT over MTX among women participating in screening [4]. Furthermore, interval cancers (detected between screenings) are shown to have a larger median tumor size, more affected axillary lymph nodes, higher proportion of grade 3, and fewer with positive hormone receptor status [5]. The selection of MTX rather than BCT could be more prominent in women with interval cancer and may be a confounding factor. Based on this, the cohort was divided into screening detection categories.

Previous studies, including a study with women participating in screening, did not examine whether there are differences in survival between BCT and MTX in early-stage breast cancer, stratified in T1N0M0, T2N0M0, T1N1M0, and T2N1M0 [1–3]. The aim of this study is to investigate, in all four stages of early breast cancer, whether there is a survival benefit when women undergo BCT compared to MTX when women participate in screening.

## Methods

A database was established when mammography screening was introduced in Norway in 1996. From this database, information on women with invasive breast cancer diagnosed from January 1998 to December 2009 was selected. Information on surgery type, tumor size, hormone receptor status, grade, histology, and TNM classification (according to the Union of International Cancer Control) [6] was merged with the national death registry containing information on cause of death.

This registry study has been performed with anonymous data, and thus, no ethical approval or consent from patients were necessary.

### Cohort selection

Treatment recommendations from the Norwegian Breast Cancer Group to accept BCT as the final result of surgery were as follows: the free margin should, from 1998 to 2003, be at least 5 and 3 mm from 2003 to 2009; an acceptable cosmetic result should be obtained; tumor size should be less than 5 cm from 2003; multifocal tumors were not accepted from 1998 to 2003; and multifocal tumors <1 cm apart were accepted for BCT from 2003. A cohort who, according to the Norwegian Breast Cancer Group recommendations, could have been offered either MTX or BCT was selected [7].

Contralateral prophylactic surgery was not recommended during the study period [7].

In Norway, women aged 50–69 years are invited to have a mammography every second year.

To evaluate possible differences in survival due to different screening detection categories, we divided the cohort into three groups: first screening (detected on the first screening), later screening (detected on second or later screening), and interval cancer (detected after normal screening and before the next scheduled screening). Only women who had participated in at least one screening were selected. A total of 8160 women aged 50–69 years with primary operable breast cancer (stages T1N0M0, T2N0M0, T1N1M0, and T2N1M0) were included and followed until the end of 2010.

Women meeting one of the following criteria were excluded: more than one infiltrating breast cancer localized in breast; multifocal (217); breast cancer not primarily located in breast (169); unknown metastasis status at diagnosis (969); metastasis at diagnosis (25); not operated (4); unknown hormone status (389); unknown nodal status (0) or unknown size of tumor in mm (0). The final cohort consisted of 6387 women. Surgery was divided into BCT and MTX as the final operation.

Hormone receptor status was regarded as positive (5449) if both (3264) or one (2185) of the hormone receptor values (ER, PrgR) were positive. Hormone receptor status was regarded as intermediate (213) if: both were intermediate (82), one intermediate and one negative (131), or one intermediate and one missing (0). Hormone receptor status was regarded as negative (725) if both were negative (722) or one negative and one missing (3).

Hormone receptor status was also stratified in estrogen-receptor (ER) positive (ER 100–10%) and ER negative status (ER < 10%).

### Statistical analysis

Life tables for overall survival (OS) and breast cancer-specific survival (BCSS, proportion of cohort who had not died of breast cancer within 5/10 years), were done in the following stages: pT1N0M0, pT2N0M0, pT1N1M0, and pT2N1M0, stratified by BCT and MTX.

Kaplan-Meier survival analyses were done on BCT and MTX, stratified in stages.

Overall death and breast cancer death figures were compared using the Cox proportional hazard model for estimating hazard ratios (HR), between BCT and MTX in crude and multivariate analyses. The multivariate analysis was adjusted for screening detection category, year of diagnosis, screening age, tumor size, nodal status, histology, grade, and hormone status. Furthermore, the adjusted analysis was stratified in stages. Sub-analysis was also done on T1-2N1M0 from year 2003 (all node positive were recommended radiation therapy from year 2003, regardless of surgical treatment). This analysis did not have enough numbers to give significant results in the T1N1M0 and T2N1M0 strata.

Statistical analyses were conducted in STATA version 13.1 (StataCorp, Texas, USA).

## Results

### Main results

In stage T1N1M0 women participating in screening who underwent MTX had a HR of 2.91 (95% CI 1.30–6.48) for breast cancer death compared to women who underwent BCT, after adjusting for screening detection, years of diagnosis, age at diagnosis, histology, grade, and hormone receptor status. In stages T1N0M0, T2N1M0, and T2N1M0, no survival benefit of BCT compared with MTX was found after adjustment.

### Baseline results

Of 6387 women diagnosed with breast cancer after participating in screening, 368 women died of all causes within 10 years of their operation. Of these, 115 died of breast cancer within 5 years. After 10 years, a total of 182 women had died of breast cancer (not in table). Median follow-up time for the whole cohort was 6.0 years (Table 1). In total, 4449 (70%) underwent BCT (Table 1) and of these 52 died of breast cancer within 5 years and a total of 88 died within 10 years of breast cancer.

**Table 1:** Baseline characteristics

	First screening		Later screening		Interval cancer		Total	
	Number 1 251 (20%)		Number 3 849 (60%)		Number 1 287 (20%)		Number 6 387 (100%)	
	Median follow-up time 8.5 years		Median follow-up time 5.6 years		Median follow-up time 5.6 years		Median follow-up time 6.0 years	
	Number (proportion)	Proportion undergoing BCT	Number (proportion)	Proportion undergoing BCT	Number (proportion)	Proportion undergoing BCT	Number (proportion)	Proportion undergoing BCT
BCT	858	69%	2 844	74%	747	58%	4 449	70%
Median size of tumor	14.2 mm		13.5 mm		18.9 mm		14.7 mm	
BCT	13.3 mm		12.5 mm		16.9 mm		13.4 mm	
MTX	16.1 mm		16.2 mm		21.7 mm		17.7 mm	
Stages								
T1N0M0	983 (72%)	71%	2 868 (75%)	80%	637 (49%)	70%	4 408 (69%)	76%
T2N0M0	102 (8%)	53%	323 (8%)	51%	282 (22%)	46%	707 (11%)	50%
T1N1M0	176 (14%)	73%	476 (12%)	65%	205 (16%)	55%	857 (13%)	64%
T2N1M0	70 (6%)	50%	182 (5%)	48%	163 (3%)	36%	415 (7%)	44%
Tumor status								
T1	1 079 (86%)	71%	3 344 (87%)	77%	842 (65%)	66%	5 265 (82%)	74%
T2	172 (14%)	52%	505 (13%)	50%	445 (35%)	43%	1 122 (18%)	47%
Nodal status								
Negative	1 005 (80%)	69%	3 191 (83%)	77%	919 (71%)	63%	5 115 (80%)	73%
1 node positive	156 (12%)	68%	469 (12%)	65%	219 (17%)	54%	844 (13%)	63%
2 nodes positive	63 (5%)	67%	127 (3%)	52%	87 (7%)	39%	277 (4%)	51%
3 nodes positive	27 (2%)	56%	62 (2%)	44%	62 (5%)	31%	151 (2%)	40%
Histology								
Ductal car.	1 030 (82%)	70%	3 136 (81%)	74%	1 020 (79%)	60%	5 186 (81%)	71%
Lobular car.	117 (9%)	56%	360 (9%)	65%	145 (11%)	48%	622 (10%)	59%
Other	104 (8%)	70%	353 (9%)	78%	122 (9%)	53%	579 (9%)	72%
Grade								
Grade1	472 (35%)	75%	1 347 (32%)	82%	278 (19%)	63%	2 097 (33%)	78%
Grade2	589 (44%)	69%	1 871 (46%)	72%	584 (44%)	61%	3 044 (48%)	69%
Grade3	161 (13%)	52%	567 (15%)	65%	411 (30%)	52%	1 139 (18%)	58%
Unknown grade	29 (9%)	45%	65 (7%)	56%	14 (7%)	21%	107 (2%)	48%
Hormone status								
Positive	1 119 (89%)	70%	3 352 (87%)	75%	978 (76%)	60%	5 449 (85%)	71%
Intermediate	44 (4%)	57%	110 (3%)	76%	213 (5%)	51%	213 (3%)	65%
Negative	88 (7%)	60%	387 (10%)	62%	725 (19%)	51%	725 (11%)	58%

### Five and 10-year breast cancer-specific survival (BCSS)

Women participating in screening had 98% (95% CI 0.98–0.99) breast cancer-specific survival (BCSS) after 5 years and 96% (95% CI 0.96–0.97) after 10 years (not in table). Both surgical groups in T1-2N0M0 had no significant difference in 5- or 10-year BCSS (Table 2). Thirteen percent of the cohort had stage T1N1M0, with significantly better survival among women undergoing BCT compared to MTX. 5-year BCSS in stage T1N1M0 was 99% (95% CI 0.97–0.99) for women undergoing BCT, and 96% (95% CI 0.92–0.98) for women undergoing MTX. 10-year BCSS in stage T1N1M0 was 97% (95% CI 0.94–0.99) for women undergoing BCT and 89% (95% CI 0.83–0.93) for women undergoing MTX. BCSS in the screening detection categories: the most favorable 10-year BCSS was 98% for women undergoing BCT in stage T1N1M0 detected on first screening and stages T1N0-1M0 detected on second or later screening. Women undergoing MTX had the following 10-year BCSS in T1N1M0 detected on first screening 93% (95% CI 74–98), 96% (95% CI 93–98) in T1N0M0 detected on second or later screening, and 91% (95% CI 82–95) in T1N1M0 detected on second or later screening. The least favorable ten-year BCSS was 64% (95% CI 0.39–0.81) in first-screening detected, stage T2N1M0 for women undergoing MTX.

**Table 2:** Survival in percentage by 5- and 10-year, stage, surgery, and screening detection group

	Detected on first screening				Detected on second or later screening				Interval cancer				Total			
	5-year		10-year		5-year		10-year		5-year		10-year		5-year		10-year	
	OS	BCSS	OS	BCSS	OS	BCSS	OS	BCSS	OS	BCSS	OS	BCSS	OS	BCSS	OS	BCSS
	95% CI	95% CI	95% CI	95% CI	95% CI	95% CI	95% CI	95% CI	95% CI	95% CI	95% CI	95% CI	95% CI	95% CI	95% CI	95% CI
All early-stages																
BCT	98 96–99	99 99–100	92 89–94	97 94–98	97 96–98	99 98–99	93 92–95	97 96–98	95 93–97	97 95–98	89 85–92	94 91–96	97 96–97	98 98–99	92 91–97	96 96–97
MTX	95 92–97	98 96–99	86 81–90	92 96–99	94 92–95	97 96–98	88 85–91	95 92–96	91 88–93	92 89–94	83 78–87	89 85–91	93 92–94	96 95–97	86 84–88	92 91–94
T1N0M0																
BCT	98 96–99	99 99–100	93 89–95	97 94–98	98 97–98	99 99–100	95 93–96	98 97–99	96 93–97	98 95–99	89 84–93	95 91–97	97 97–98	99 99–99	93 92–95	98 97–98
MTX	95 92–97	99 96–100	87 82–91	94 90–97	94 92–96	98 96–99	89 85–92	96 93–98	97 93–99	100 –	91 83–95	96 89–99	95 94–96	99 98–99	89 86–91	96 93–97
T2N0M0																
BCT	98 86–100	98 86–100	94 77–99	94 77–99	94 88–97	95 90–98	85 73–91	88 77–94	92 84–96	94 88–96	87 77–93	92 83–96	94 90–96	95 92–97	87 81–92	90 85–94
MTX	100 –	100 –	93 74–98	100 –	92 86–96	97 92–99	83 74–90	94 86–97	86 78–91	86 79–91	81 72–88	85 77–90	91 87–93	93 89–95	84 78–88	91 87–94
T1N1M0																
BCT	98 93–100	99 94–100	88 78–94	98 90–99	98 95–99	99 96–99	94 95–99	98 96–99	97 90–99	97 90–99	92 81–97	94 85–98	98 97–99	99 97–99	92 88–95	97 94–99
MTX	96 83–99	100 –	86 68–94	93 74–98	93 88–95	96 90–98	87 78–93	91 82–95	92 83–96	93 85–97	79 65–88	85 72-93	93 90–96	96 92–98	84 78-89	89 83–93
T2N1M0																
BCT	94 77–98	97 80–100	88 65–96	91 65–98	89 79–94	94 86–98	73 79–94	81 86–98	96 84–99	98 85–100	81 57–92	87 63–96	92 86–95	96 91–98	79 68–86	85 75–91
MTX	84 66–93	87 69–95	62 38–79	64 39–81	95 86–98	96 87–99	95 86–98	96 88–99	85 75–90	86 76–92	75 62–70	83 72–82	88 83–92	90 85–95	80 71–86	84 77–90

### Screening detection category

Screening detection of cancer was distributed as follows: first screening 20% (1251), later screening 60% (3849), and interval cancer 20% (1287) (Table 1). The highest proportion of women who underwent BCT within the screening categories was found among the later screening group (74%), and the lowest in interval cancer (58%).

### Kaplan-Meier curves

**Fig. 1:**
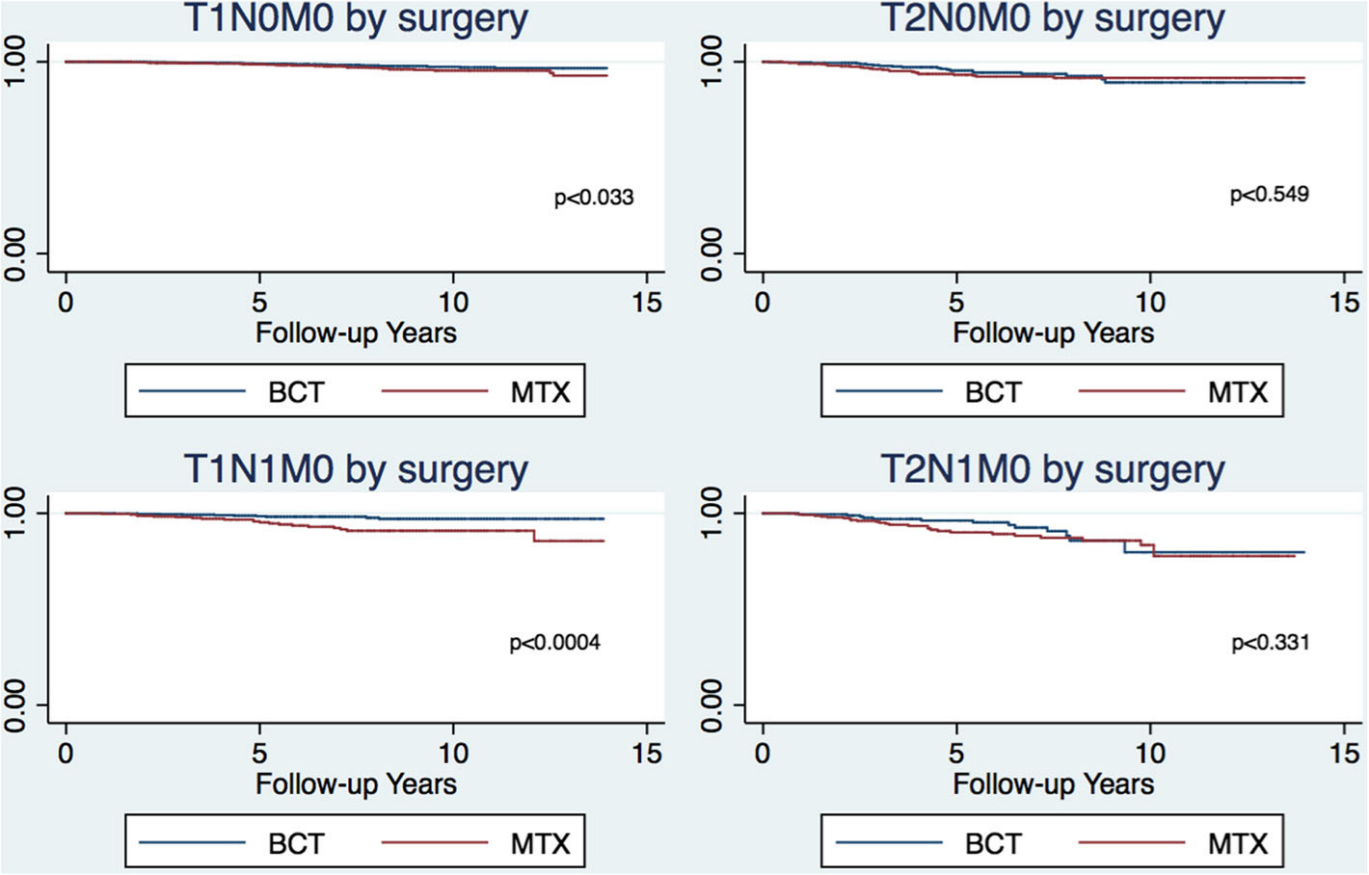
Kaplan-Meier survival analysis stratefied by TNM stages and surgery

Corresponding Kaplan-Meier curves show the benefit of BCT over MTX in stage T1N1M0 (Fig. 1). Furthermore, the Kaplan-Meier curves also show the benefit of BCT over MTX in stage T2N1M0 after 5 years, but at 8 years, the curves align.

### Crude and adjusted analyses

Crude HR for breast cancer death for women undergoing MTX compared with BCT was 2.33 (95% CI 1.75–3.10) (Table 3). In the adjusted analysis, HR for breast cancer death for women undergoing MTX compared with BCT was 1.39 (95% CI 1.02–1.89). Adjusted analysis on breast cancer death gave interval cancer HR 1.32 (95% CI 0.87–2.00) compared with HR 1.0 detected on first screening. In the stratified adjusted analysis on stage T1N1M0, the HR for breast cancer death was 2.91 (95% CI 1.30–6.48) for women undergoing MTX compared with BCT with HR 1.0 (main result, Table 4). The same stage adjusted by tumor size in mm resulted in HR 3.13 (95% CI 1.32–7.45) for women undergoing MTX compared with BCT with HR 1.0 (result not shown in table). Furthermore, the same analysis, done with hormone receptor status divided into estrogen-receptor positive or estrogen-receptor negative status, resulted in HR for breast cancer death 2.69 (95% CI 1.21–6.00) compared with BCT with HR 1.0. In a stratified adjusted analysis on stage T1N0M0, T2N0M0, and T2N1M0, no survival benefit of BCT compared with MTX was found. Sub-analysis on stage T1N1M0 and T2N1M0 from year 2003 (women in both surgical cohorts with node positive disease were recommended radiation therapy) resulted in HR for breast cancer death 2.25 (95% CI 1.21–4.17) for women undergoing MTX compared with BCT (result not in table).

**Table 3:** Overall deaths/breast cancer deaths, crude and adjusted comparing BCT to MTX

	Crude		Adjusted	
	Overall death (95% CI)	Breast cancer death (95% CI)	Overall death (95% CI)	Breast cancer death (95% CI)
Surgery (adjusted stage)				
BCT	1	1	1	1
MTX	1.91 (1.56–2.33)	2.33 (1.75–3.10)	1.43 (1.16–1.77)	1.39 (1.02–1.89)
Screening				
First screening	1	1	1	1
Second or later	0.96 (0.74–1.24)	0.88 (0.60–1.30)	0.91 (0.69–1.18)	0.90 (0.61–1.36)
Interval cancer	1.65 (1.25–2.18)	2.39 (1.62–3.52)	1.12 (0.83–1.50)	1.32 (0.87–2.00)
Year of diagnosis				
1998–2004	1	1	1	1
2005–2009	0.58 (0.45–0.75)	0.49 (0.34–0.71)	0.59 (0.45–0.76)	0.48 (0.33–0.70)
Screening age				
50–53	1	1	1	1
54–57	1.65 (1.13–2.41)	1.60 (1.00–2.59)	1.69 (1.15–2.49)	1.62 (0.99–2.64)
58–61	1.80 (1.24–2.59)	1.45 (0.90–2.34)	1.95 (1.34–2.84)	1.65 (1.01–2.69)
62–65	1.94 (1.34–2.88)	1.26 (0.77–2.09)	2.18 (1.49–3.18)	1.47 (0.88–2.46)
66–69	2.29 (1.58–3.32)	1.26 (0.74–2.13)	2.61 (1.78–3.82)	1.59 (0.93–2.72)
Tumor size				
T1 ≤ 2 cm	1	1	1	1
T2 > 2–5 ≤ cm	2.27 (1.83–2.81)	3.86 (2.90–5.15)	1.52 (1.22–1.97)	2.08 (1.51–2.86)
Nodal status				
Negative node	1	1	1	1
1 positive node	1.45 (1.10–1.90)	2.09 (1.46–2.99)	1.32 (1.00–1.74)	1.65 (1.15–2.38)
2 positive nodes	1.70 (1.14–2.52)	2.48 (1.49–4.12)	1.33 (0.89–1.99)	1.55 (0.92–2.62)
3 positive nodes	2.39 (1.49–3.85)	3.68 (2.03–6.66)	1.67 (1.02–2.72)	1.96 (1.06–3.63)
Histology				
Ductal carcinoma	1	1	1	1
Lobular carcinoma	0.77 (0.53–1.13)	0.73 (0.42–1.26)	0.77 (0.52–1.14)	0.85 (0.48–1.49)
Other carcinoma	0.96 (0.68–1.36)	0.89 (0.53–1.49)	0.96 (0.67–1.36)	0.83 (0.49–1.40)
Grade				
Grade1	1	1	1	1
Grade2	1.57 (1.21–2.03)	2.48 (1.57–3.91)	1.34 (1.02–1.76)	1.83 (1.15–2.92)
Grade3	3.05 (2.31–4.04)	7.58 (4.81–11.93)	1.66 (1.19–2.31)	2.91 (1.73–4.88)
Unknown grade	1.17 (0.51–2.69)	4.64 (no value)	0.84 (0.36–1.96)	Missing
Hormone status				
Positive	1	1	1	1
Intermediate	1.66 (1.05–2.61)	3.61 (2.17–6.03)	1.41 (0.89–2.24)	2.48 (1.47–4.20)
Negative	2.83 (2.24–3.58)	5.01 (3.68–6.82)	1.98 (1.50–2.62)	2.61 (1.80–3.79)

**Table 4:** Overall deaths/breast cancer deaths, crude, and adjusted

	Crude		Adjusted	
	Overall death (95% CI)	Breast cancer death (95% CI)	Overall death (95% CI)	Breast cancer death (95% CI)
MTX				
T1N0M0	1.67 (1.27–2.21)	1.65 (1.04–2.62)	1.47 (1.11–1.96)	1.32 (0.83–2.12)
T2N0M0	1.42 (0.86–2.32)	1.20 (0.66–2.20)	1.46 (0.86–2.42)	1.26 (0.68–2.33)
T1N1M0	2.48 (1.45–4.24)	3.61 (1.69–7.72)	2.08 (1.19–3.64)	2.91 (1.30–6.48)
T2N1M0	1.09 (0.63–1.90)	1.39 (0.71–2.72)	1.19 (0.66–2.15)	1.40 (0.69–2.86)

The adjustments are done by screening detection category, years of diagnosis, screening age, nodal status, histology, grade, and hormone status (same as Table 3). BCT is base 1.0

## Discussion

The most important finding in this study is the benefit of BCT compared with MTX in stage pT1N1M0. In all other early stages of breast cancer, there were no significant benefit of BCT over MTX when women participate in screening. In a previous study on women participating in screening, adjusted analysis revealed a 1.7 (95% CI 1.3–2,4) higher risk of breast cancer death among women who underwent MTX compared with BCT [4]. However, this study did not do the adjusted analysis in different stages of early breast cancer.

Previous registry studies have shown better survival among women undergoing BCT compared with MTX, the latest published from the Netherlands with 20 years of follow-up time [1–4, 8]. A recently published Danish study has shown better overall survival among women aged <45 undergoing MTX compared to BCT. However, women aged ≥45 had significant better overall survival when they underwent BCT compared to MTX [9]. In these studies, the adjusted analysis was done by size, nodal status, or stage [1–3, 8]. In the present study, the adjusted analysis was also done after stratifying by stage and screening detection category. The finding of better survival rates among women undergoing BCT compared with MTX when there are positive nodes in the axilla might contribute to identifying where to find the cause of the difference in survival between women undergoing BCT and MTX.

Studies on small (T ≤ 1.5 cm), lymph node-negative breast tumors show very high breast cancer survival rates at 10 years, even in the absence of chemotherapy [10]. In this study, 5-year BCSS in stage T1N0M0 is 99% in both surgical groups.

Since better survival rates among stage T1N1M0 were found, improved survival among those with a larger tumor (T2N1M0) could have been expected. Crude and adjusted analyses do not show any significant benefits of BCT over MTX in stage T2N1M0. However, the Kaplan-Meier curve shows an advantage of BCT over MTX until 8 years of follow-up. In this cohort, only 376 had T2N1M0—a study with a larger cohort might find a significant benefit of BCT over MTX within the T2N1M0 strata. Furthermore, if there had been a strong benefit from BCT compared with MTX, this would probably have been shown in earlier studies. Clinical trials comparing BCT with MTX done decades ago show similar survival benefits of BCT and MTX [11–16]. Since these studies were conducted, treatment has changed and survival improved [7, 17].

## Limitations in the study

### Tumor size

Tumor size was slightly larger in women undergoing MTX compared with BCT. In Hwang et al.’s and Agarwal et al.’s studies, tumor size was analyzed by size groups defined in cm. This could be a confounding factor; however, the adjusted analysis in this study on tumor size in mm, within stage T1N1M0 did not reduce the HR between MTX and BCT. Small differences in tumor size hardly explain the difference in survival between BCT and MTX in stage T1N1M0.

### Nodal status

The number of positive nodes involved might differ between the surgical groups. For instance, a larger proportion of positive lymph nodes in women undergoing MTX compared with a low proportion of positive lymph nodes in women undergoing BCT would probably contribute to a difference in survival between BCT and MTX [18, 19]. This is the reason why the adjusted analysis was done by number of nodes involved and not by the TNM classification of nodal status (N0 or N1).

### Screening detection categories

Dividing the cohort into screening detection categories was done based on an assumption that cancer detected on first screening could have different clinophatological features compared to later screening-detected cancer and interval cancer. Furthermore, selection toward MTX compared with BCT could be more prominent in women with interval cancer and may be a confounding factor. A study on interval cancer in Norwegian breast cancer screening done in 2001 [5] showed that interval cancer had a higher proportion of larger tumors, affected axillary lymph nodes, grade 3, and hormone-negative disease. These results are comparable with our results, where interval cancer tumors were larger and had a higher proportion of affected axillary lymph nodes, and grade 3 compared to screening-detected cancer. Crude breast cancer death is significantly higher among women with interval cancer compared to screening-detected cancer. However, in the adjusted analysis, there are no significant differences in breast cancer death between the first-screening, later-screening, and interval-screening groups. Based on this, it is unlikely that a selection toward MTX among women with interval cancer can explain the difference in survival between BCT and MTX.

### Follow-up time

A high proportion of breast cancers are detected with only one mammography performed, the first screening. This is why the first screenings have the longest median follow-up time.

### Grade and hormone status

Survival benefit decreases with higher-grade classification [20] and negative hormone receptor status. Both grade and hormone status are taken into account in the adjusted analysis. Furthermore, a sub-analysis on hormone receptor status divided only into estrogen-receptor positive or estrogen-receptor negative status showed no significant difference compared to the analysis with positive, intermediate, or negative hormone status.

### Adjuvant therapy

Recommendations of antiestrogen therapy and chemotherapy are the same in both surgical groups and very standardized in Norway [7]. This is the first registry study showing equal survival benefit between women undergoing BCT or MTX in stage T1N0M0, T2NM0, and T2N1M. If the findings in this study were due to a difference in adjuvant therapy, differences in all stages would probably also be found. However, no details on the adjuvant therapy given were available, and studies with details on this are needed.

### Choice of surgery

A study on why MTX rates vary found that women recalled less autonomy and less time for decision-making when treated in a breast unit with a low proportion of MTX than women treated in unit with a high proportion of MTX [21]. Conversely, women from the high and medium MTX rates units described provision of more comprehensive less directive information, together with greater support and time for more autonomous decision making. In brief, the selection towards MTX seems to be the women’s choice. Other studies support this finding [22–24].

One study that might have influenced the surgeon’s recommendation towards MTX in the later study period was published in 2005 [25]. Interpretation of this study was avoidance of one breast cancer death over the next 15 years for every 4 local recurrences avoided.

However, during study period, BCT and MTX were considered to give equal survival benefit when contraindications for BCT were followed [7]. This included obtaining free margins and tumor size less than 5 cm. (median tumor size in total study cohort is 14.7 mm). All women with multifocal tumors were excluded. Grade, hormonal, and nodal status (N0 or N1) did not influence guidelines regarding selection of surgery. Furthermore, if this had an influence in the clinical setting, these factors are taken into account in the adjusted analysis.

Sentinel node biopsy (SNB) was introduced in Norway in 2000. The indications for doing SNB were the same in both surgical groups during the study period.

### HER2 and Ki67

Human epidermal growth factor receptor 2 (HER2) was recommended as a routine examination in 2005, late in the study period [7]. Therefore, diagnosis year was grouped into 1998–2004 and 2005–2009. Based on this, it is unlikely that a difference in trastuzumab treatment explains the difference between the surgical groups. Furthermore, no tumor biology markers determine recommendations on surgery type [7]. The proliferation marker Ki67 was not measured during the study period.

### Radiation therapy

All women undergoing BCT were recommended RT. Recommendations on radiation therapy (RT) regarding the nodal status changed during the study period. From 1998, all women aged <55, undergoing MTX with less than 4 positive lymph nodes, were not recommended radiation therapy. From 2000, all women aged <55 with 1–3 positive lymph nodes were recommended radiation therapy. From 2003, all women aged <70 years were recommended RT if 1–3 lymph nodes were positive. Sub-analysis was therefore performed on all women with stage T1-2N1M0 from 2003 (all women undergoing MTX with N1 status were recommended RT). This sub-analysis showed the benefit of BCT compared with MTX. Based on this, RT does not seem to be the main reason for the survival benefit seen in T1N1M0 in this study. It might be a combination of BCT and RT that improves survival and not RT alone. On the other hand, a meta-analysis of 8135 women published in 2014 showed the survival benefit of radiation therapy after mastectomy and axillary dissection [26]. A reduced use of RT in the MTX group might, to some degree, favor the BCT group.

## Alternative explanations of findings

### Surgery

Some studies have suggested that the extent of surgery can be a negative factor regarding survival. In a study by Cheng K.et al. [8], they refer to studies on animal models in which it is suggested that the surgical trauma of normal tissue promotes the implantation or growth of circulating tumor cells [27–30]. A recently published study regarding recurrence pattern following delayed breast reconstruction after MTX for breast cancer suggests a systemic effect of surgery on occult dormant micro metastases [31].

### Immune response

Doxorubicin is shown to increase the tumor antigen-specific proliferation of CD8 T cells in mice with carcinogen-induced tumors [32]. There is accumulating evidence that some cytotoxic drugs, such as taxane, actually promote antitumor immunity and thereby contribute to the treatment’s therapeutic effect [33]. Women who have undergone BCT might have a better immune response against breast cancer cells compared with those who have undergone MTX. As a hypothesis, RT against remaining satellite tumors in the conserved breast with following necrosis of tumor tissue enhances an improved immune response against the cancer. A similar hypothesis might be introduced for the combination of BCT and chemotherapy and even the combination of BCT, chemotherapy, and RT. As far as we know, this hypothesis has not been tested.

## Conclusions

The most important finding for women participating in screening is that there is a survival benefit of BCT compared with MTX in stage T1N1M0, but no other early stages of breast cancer.

## Abbreviations

BCSS: Breast cancer-specific survival
BCT: Breast-conserving therapy
HR: Hazard ratios
MTX: Mastectomy
OS: Overall survival
